# Reduction of Cracks in Marble Appeared at Hydro-Abrasive Jet Cutting Using Taguchi Method

**DOI:** 10.3390/ma15020486

**Published:** 2022-01-09

**Authors:** Sorin Barabas, Adriana Florescu

**Affiliations:** Faculty of Technological Engineering and Industrial Management, Transilvania University of Brasov, 500036 Brasov, Romania; sorin.barabas@unitbv.ro

**Keywords:** marble processing, hydro-abrasive jet cutting, reduction of cracks, method of Taguchi fractional experiment plans

## Abstract

The appearance of cracks in brittle materials in general and in marble, in particular, is a problem in the hydro-abrasive jet cutting process. In this paper is presented a method to reduce the appearance of cracks when cutting with a hydro-abrasive jet of marble by using statistical analysis. The Taguchi method was used, establishing the main parameters that influence the process. Research design was based on performing experiments by modifying the parameters that influence the process. In this way, it has been shown that the stochastic effects resulting from the marble structure can be reduced. A careful study was made of the behavior of marble under the action of the hydro-abrasive jet, and of the behavior of the whole process in the processing of brittle materials. Results of experiments confirmed the hypothesis that statistical analysis is a procedure that can lead to a decrease in the number of cracks in processing. The measurement was performed with precise instruments and analyzed with recognized software and according to the results obtained, the reduction of the number of cracks is achieved through use of low pressure, a minimum stand-off distance and a small tube diameter. In this way, the paper presents a new and effective tool for optimizing the cutting with a hydro-abrasive jet of marble.

## 1. Introduction

The development of hydro-abrasive jet cutting technology has led to its application in all processing sectors, to greater accessibility, to high-performance machines. Thus, jet machines began to be included in any larger or smaller manufacturing unit. Basically, the technology of abrasive jet cutting has become widespread. Equipment manufacturers invest heavily in research into this successful technology, keeping it at the forefront of unconventional technologies. The results of recent research are seen in the increase of the performances in the working speed, the increase of the quality, versatility and the decrease of the price of the products [1,2,3]. The possibilities of machines configuration are greater and use has become easier. Thus, the users of the technology are put in front of many options for choosing the necessary equipment as well as the great configuration possibilities. These, as well as the type of small and unique series production, made on the abrasive jet cutting machines led to the appearance of tools for optimizing the whole process. In this paper, such a tool is proposed for optimizing the jet processing of brittle materials, especially marble.

The abrasive jet cutting process is based on the detachment of particles from the processed material by breaking, on impact with the abrasive particles of the jet. In the case of brittle materials, there is a danger of the development of cracks or microcracks in the impact area, their appearance being able to be eliminated by a correct configuration of the working parameters. The material on which the experiments were performed was marble. Only the appearance of microcracks was taken into account, and as an objective it was taken to decrease their number over a linear processing distance. The appearance of cracks in brittle materials is a danger in the manufacturing process because they have an unpredictable character determined by the many characteristics of the manufacturing process and the properties of the material used. Even taking into account these elements there are many other possibilities to prime the cracks: the working environment, the position of the crystals in the processed material, defects of the material, the defective grip of the parts being thus impossible to prevent the phenomenon accurately. The way to reduce the number of cracks is to use statistical methods, by conducting experiments and interpreting the results. Starting from the hypothesis these methods applied to the elements that can be modified, namely the cutting regime, the type of abrasive, the type of nozzle and the distance from the part will lead to the reduction of the number of cracks. This research aims to demonstrate that through a judicious choice of working parameters, they can be configured in such a way as to reduce the number of cracks and microcracks in the brittle parts. Thus, the following controlled parameters were considered in the configuration of the hydro-abrasive jet cutting process: pressure P (MPa), type of abrasive (garnet 80 or garnet 100), nozzle diameter D (mm) and distance between nozzle and piece (stand-off distance) d (mm). Many researchers have studied the phenomenon of material removal and the appearance of micro-cracks in brittle materials.

Melentiev et al. [4] investigate the phenomenon of chipping from abrasive materials at the action of the abrasive jet, studying the importance of the type of material and the geometry of the nozzle, as well as the importance of the abrasive, concluding that the mechanism of material removal on brittle materials cannot be fully clarified without taking into account of processed material random structure (position of the grains, connection between them, etc.).

The subject was researched under different aspects by Arab et al. [5], noting the importance of material characteristics homogeneity, porosity, inclusions, granulation, pre-existing microcracks, making a comparison between different types of rocks, granite, marble, syenite and sandstone and their tendency to crack.

The distribution of jet abrasive particles and their stochastic action on materials in the formation of cracks has also been intensively studied, Torrubia et al. [6] use finite element analysis, Pawar et al. [7] uses multi-objective artificial bee colony algorithm, Rao et al. [8] in 2017 uses Jaya algorithm, Perec [9] in 2021 models and optimizes the abrasive jet cutting process using the method of response surfaces. Natarajan et al. [10] in 2019 make a detailed study on the effect of the stand-off distance, as well as the type of abrasive used. Liu et al. [11] in a review of hydro-abrasive jet technology in 2019 explain the process of chip removal for brittle materials and determining the probability of cracks using adaptive neural fuzzy network systems. The authors propose the method of crack reduction, a polishing with a hydro-abrasive jet. Zheng and He in 2021 [12] studies the cutting of brittle materials with the help of an abrasive disc assisted by two hydro-abrasive jets.

A recent research study [13] proposes an approach to the complex process of cutting hard rock, analyzing and evaluating the intensity of the disintegration of abrasive materials in the process of cutting AWJ, during the creation of the abrasive jet. Some current processing models on abrasive water jet machines used in the analysis of the behavior of various materials, including rock type are presented in the papers [14,15,16], but in the field of marble cutting by AWJ process research is limited.

Relevant research that reflects current trends in the field of abrasive water jet cutting of various materials was conducted in the study [17], highlighting the functions and influences of the parameters of the cutting process. An extensive review presents the statistics of the various materials to be processed (from metallic materials, composite materials to natural materials) used in AWJM applications, in a series of recent publications, revised from 2017–2021. It can be seen that at present, in the field of cutting natural materials, such as marble or rock, there are very few research studies [4,18] that highlight the quality problems and defects strongly influenced by the properties of these materials. Hlaváč et al. [19] presents a new analytical model for evaluating abrasive water jet cutting, and presents some characteristics of hard rock materials (marble, limestone, granite), without specifying the quality defects that occur in processing surfaces, much less methods of reducing them. This paper will address the gaps in existing studies on the processing of rock (marble) materials on abrasive water jet cutting machines, taking into account the characteristics of these materials.

The complexity of the whole system determines the variability of the results randomly and the best way in optimizing the results is to use statistical methods. Analysis of variance, ANOVA, is a statistical tool that takes into account the contribution of each parameter that acts on the system, distribution of results being directly related to the action of each factor. The necessary experiments must take into account the variation of each parameter resulting in too many samples. A method that significantly reduces the number of samples is the method of fractional testing by using orthogonal matrices of experiments (Taguchi method). This method has been used in the optimization of the jet cutting process by many authors in order to increase the dimensional accuracy and improve the quality of the resulting surfaces Gupta et al. [20], Joel and Jeyapoovan [21], Perumal et al. [22], Balaji et al. [23], Barabas and Deaconescu [24].

This paper aims to study the reduction of the number of cracks in marble during hydro-abrasive jet processing, using statistical methods. The method of studying the results is the method of fractional experimental plans.

## 2. Theoretical Considerations

### 2.1. The Process of Creating and Propagating Microcracks in Marble

Marble is a metamorphic rock obtained from limestone under conditions of high pressure and temperature. It is most often composed of calcite—CaCO_3_ or dolomite—CaMg(CO_3_)_2_, by recrystallization. The main properties of marble are: porosity (%), density (g/cm^3^), compressive strength (MPa), flexural strength (MPa), Amsler abrasion wear (mm/1000 m), hardness (Mohs), Young’s modulus (GPa), fracture toughness (MPa/m) [2,14] and largely depend on the area from which it comes.

**Figure 1:**
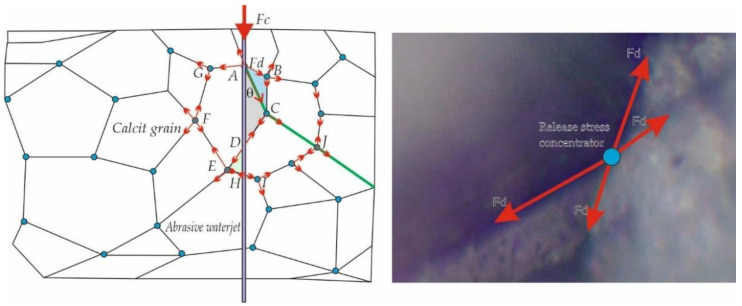
The model of propagation of the separation forces of the grains in marble, under the action of an external cutting force.

Appearance of bifurcated cracks and microcracks as a result of the dynamic fracturing that accompanies the rupture of the marble particles under the action of the abrasive jet, appears in all three directions of space favoring the fracture of the material. Distribution of cracks in brittle materials was studied by Anstis et al. [25], Lawn [26], Le et al. [27] and Conti [28]. By introducing the stress coefficient k, they established that the main direction of propagation, located at a q angle is on the direction of application of the cutting force and the increase of the crack propagation speed determines the appearance of secondary cracks, propagated on lateral directions (Figure 1).

At the moment of applying the abrasive jet, the garnet grains of abrasive waterjet hit the marble, generating a force F_c_ which, by cutting, forms a channel with the minimum thickness of the abrasive jet. When the jet reaches the limits of the marble grains, the shear force Fc is distributed in the lateral planes in the direction of the connecting planes between the marble grains, with a value F_d_ = F_c_ · cosθ.

Thus, a separation force F_d_ appeared, spreads along the separation planes until it meets another marble grain where it redistributes its components along the new planes encountered. Points A, B, C…. are points of redistribution of the force of separation. It can be observed, according to Figure 1, that the detachment force is inversely proportional to the value of the angle θ, it becoming the maximum when the angle θ tends to 0, and the minimum when the angle θ tends to 180°, and it will increase again. Thus, the ABC, ACD and DEH grain are most affected by the separation force F_d_. Also, the ACI and DE propagation directions are the most affected. In reality, the distribution and shape of the grains, the quality of the connections between the marble grains are random, generating the possibility of its cracking.

Taking into account that the material used in the hydro-abrasive jet cutting experiments was white Ruschita marble, formed by recrystallization, mainly from calcite, presenting a coarse crystalline structure made of crystals interconnected to each other, favors the triggering of fractures. The connection between the crystals, the position of the sliding planes, the alignment of the crystals, the foliation and the thickness of the layers formed by metamorphism, determine the mechanical properties of the marble, the resistance, the elastic modulus, the Poisson’s ratio, the thermal expansion, etc.

The most likely place for cracking is the separation limit of the crystals or inclusions contained. They propagate along the separation plane and when it meets another plane it bifurcates and expands depending on the size of the force that is applied. When the crack propagation energy, which depends directly on the application force, is large enough, a major crack occurs. If the crack does not meet sufficient resistance in the direction of propagation due to the adhesion between the crystals and the position of the sliding planes, the crack leads to the rupture of the material. Thus, the appearance of cracks depends on factors that can be controlled and factors that cannot be controlled that intervene on the system stochastically. Among the controlled factors, can be listed the factors related to the construction of the processing equipment, namely: capacity factors (precision, repeatability, rigidity), constructive factors (number of processing axes, type of focusing tube, orifice diameter, mixing chamber sizing), work (pressure, cutting regime, mass flow). Also in the category of controlled factors are the factors adjacent to the processing (type of abrasive, distance of the part—nozzle).

In the case of cracks in marble in particular and in brittle materials in general, during abrasive jet processing, uncontrolled factors are multiple: uncontrolled factors related to the processing process (variability of supply elements to the external network, wear on processing elements, working environment humidity, particle shape), uncontrolled factors related to the properties of the processed material (porosity, strength, inclusions, position of grain sliding planes, their shape, hardness, elastic modulus) and subjective uncontrolled factors (experience, fatigue, inattention).

The total number of predictable and unpredictable elements that can act on the processing process to trigger the cracking phenomenon is large. Therefore, the only way to control cracking remains statistical analysis. The application of statistical analysis is the subject of this research.

### 2.2. The Process of Cutting Brittle Materials with Abrasive Jet

The working principle of hydro-abrasive jet processing is based on the transformation of water brought at high pressures (200–700 MPa) into a high speed water jet (approx. 1000 m/s), obtained by passing water through a small orifice size (approx. 0.1 mm). On impact, the jet, consisting of air particles, water and abrasive, hit the piece, detaching the micro-chips from the material. The cutting mechanism depends on the type of material processed, distinguishing the mechanism in the elastic, plastic and brittle area [29].

**Figure 2:**
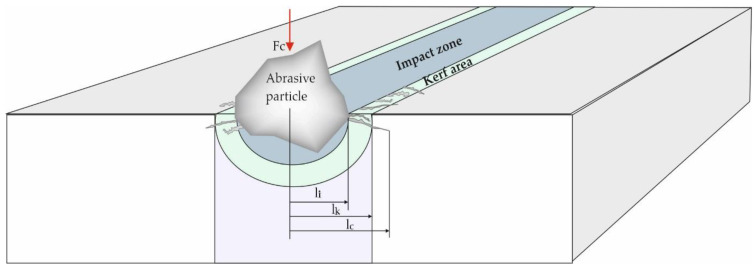
The mechanism of hydro abrasive erosion for brittle materials.

Jafar [30], determines the main elements of the erosion mechanism for brittle materials, introducing three affected areas: the impact area, the kerf area and the possible cracking area (Figure 2). In 2015, together with Jafar et al. [31], dimensioned these areas, showing that the contact area is an area to which the laws of plastic deformation are applied. Dun et al. [32] study and explain the phenomenon of the appearance of the kerf zone. Thus, the particles in the abrasive jet hit the piece, detaching the chips from the material and together are directed backwards, hitting sideways in the formed channel, widening it. Thus, the material cracks vertically under the effort of the impact force when the flow resistance of the material is exceeded. At this point, the bifurcated crack propagation process begins. At the moment of the impact between the particles of the hydro-abrasive jet with the piece, a compressive force develops that acts on the hit area, determining a plastically deformed region. With the formation of the cut channel in the cutting area, a main vertical crack appears with branches and bifurcations in the lateral area (Figure 2).

The elements that influence the size of these cracks, described in Section 2.1, determine the volume of the remote area. Considering the radius of the remote volume, one can write [31],

(1) $$h=\sqrt[{3}]{\frac{3K}{2\pi H}}$$

where *h* represents the radius of the hemisphere approximating the contact area [mm], *K* [J] represents the kinetic energy of the abrasive particle and *H* [Pa] is the hardness of the material in Vickers scale.

Slikkerveer et al. [33] use Hill’s theory of anisotropic plastic deformations and propose the size of the compressed, plastically deformed area, with l_k_ [mm].

(2) $$l_{k}=0.63h\sqrt{\frac{E}{H}}$$

where *H* represents the hardness of the Vickers material [Pa], and *E* the modulus of elasticity [Pa].

**Figure 3:**
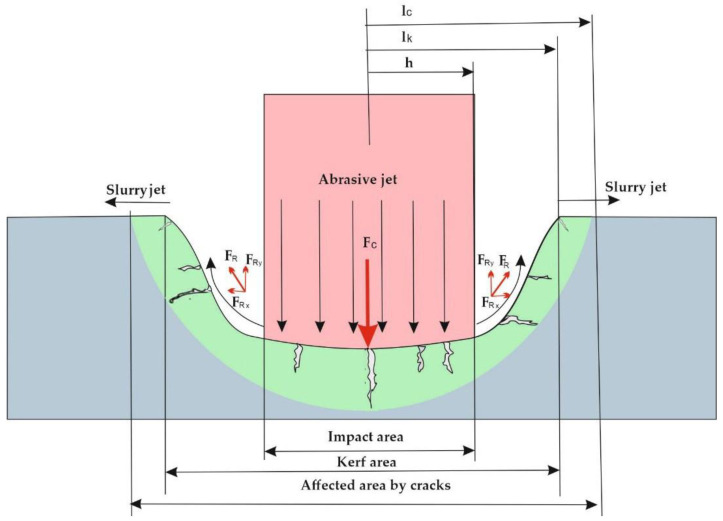
Action of slurry jet on lateral direction.

About a kerf zone, Dun et al. [32] study the behavior of the particles during and after the impact, these being rejected at the moment of initiating the cutting operation with a repulsive force *F_R_* (Figure 3)_._ The *F_R_* component along the *X* axis marked with *F_RX_* is practically the force that widens the impact zone forming the kerf area with a radius *l_k_*. Considering *P_x_* the force with which a single particle acts in the *X* direction and *d_p_* the diameter of that particle, the force exerted by the slurry jet on the lateral direction will be:

(3) $$F_{RX}=\overset{n}{\underset{i=1}{\sum }}\frac{\pi d_{pi}^{2}}{4}P_{xi}$$

The mentioned models in which the behavior of the cut material and of the abrasive particles are ideal are valid in the researched conditions. In reality, the behavior of the hydro-abrasive jet cutting phenomenon of brittle materials depends on many variables. The behavior of the abrasive particles depends on the thickness of the cut layer, the particles act differently depending on the depth they reach. Zhang et al. [34] show that before leaving the material, the abrasive particles tend to hit the workpiece in the side wall of the cut area, increasing the risk of lateral cracks and even tearing of the material. Qu et al. [35] link the behavior of particles related to their type and the type of processed materials. The behavioral variety of the particles, of their shape and type, of the processing conditions makes it impossible to predict by mathematical calculation exactly the size of the discussed areas (Figure 3). The sizing of the area affected by cracks can only be achieved starting from different working hypotheses.

Hutchings et al. [36] calculate the length of the cracks, *l_c_* [mm], as a function of the force exerted by the particle on the material:

(4) $$l_{c}=\alpha \frac{{\left(\frac{E}{H}\right)}^{\frac{3}{5}}F_{RX}{}^{\frac{5}{8}}}{\sigma_{r}^{\frac{1}{2}}H^{\frac{1}{8}}}$$

where *F_RX_* is the loading force of the particle [N], E is the modulus of elasticity [Pa], *H* represents the hardness of the Vickers material [Pa], *σ_r_* represents the breaking force of the material [N] and *α* is a coefficient determined by the particle shape of abrasive.

The volume of material displaced in the case of a jet of N particles becomes:

(5) $$Q=\beta N\frac{\left(\frac{E}{H}\right)F_{RX}{}^{\frac{9}{8}}}{\sigma_{r}^{\frac{1}{2}}H^{\frac{5}{8}}}$$

where *Q* represents the volume of displaced material [mm^3^], *N* represents the number of particles in contact with the part, and β is a coefficient that takes into account the properties of the processed material.

The equipment for processing with a hydro-abrasive jet of the brittle materials are provided from the factory with the option of low pressure. This option is available to users and is widely used but is not enough to get the best results. In this sense, the distance of the nozzle-workpiece, the diameter of the mixing tube and the type of abrasive have an equally important role. The optimization of the processing in the sense of reducing the number of cracks can be done only by choosing the optimal variants of these sizes that influence the process.

This research aims to observe the effect of each of the factors mentioned above on the appearance of cracks in marble processing and to offer users values of these factors as well as indications for optimizing the process of cutting with hydro-abrasive jet in order to reduce the number of cracks.

## 3. Materials and Methods

The material used in the experiments they performed was white Ruschita marble, a marble whose properties are similar to Carrara marble, with a density of ρ = 2710 kg/mm^3^, porosity of Φ = 0.58%, compressive strength σ_c_ = 110 N/mm^2^. The white Ruschita marble was formed by metamorphosing the limestone, at high heat and pressure, the calcite recrystallizing in a hexagonal system, in a structure of interconnected crystals presenting a great variety of shapes.

**Figure 4:**
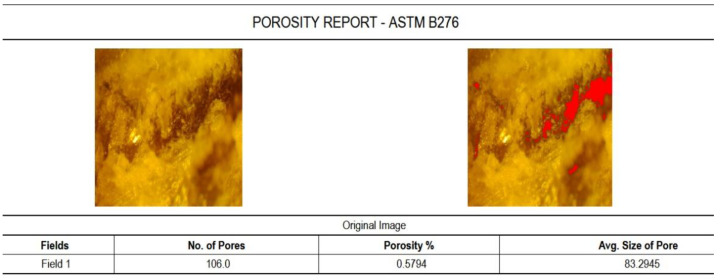
The report of porosity for Ruschita marble made with Material Plus software.

Using Material Plus 4.2 software, the samples of marble were investigated regarding porosity and inclusions (Figure 4).

As it appears from the analyzes, the marble has a good porosity (Figure 4) and a small number of inclusions (Table 1).

The tests were performed on the Maxiem 1530 hydro-abrasive jet cutting machine equipped with numerical control, using Intelli-Max Make software. The water pressure achieved with a direct drive pump was chosen having the values P_1_ = 180 MPa, P_2_ = 220 MPa and P_3_ = 250 MPa, configured by the machine software. Furthermore, 2 jet directing tubes were used, the first having a diameter D1 = 0.75 mm and the second D2 = 0.25 mm.

The abrasive used was garnet with a grain size G_1_ = 80 Mesh and G_2_ = 120 Mesh respectively, conform Table 2.

**Figure 5:**
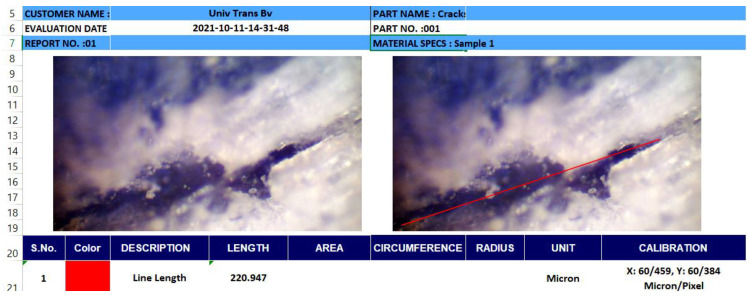
Crack measurement with Material Plus software.

To reduce the number of cracks in the marble after abrasive cutting, the parameters that influence the process were chosen first, parameters that can be modified from a technical point of view. The choice of parameters was made both in terms of possibilities and in terms of influence demonstrated so far. Thus, the pressure P [MPa], the stand-off distance d [mm], the type of abrasive and the type of nozzle (diameter) D [mm] were chosen as factors that can influence the appearance of cracks. The optimization of the marble cutting process is completed by choosing the optimal values of these parameters. This is done using statistical methods, specific to robust system design. Thus, in this research, the method of Taguchi fractional experiment plans will be used. The aim is to minimize the number of cracks in the marble. The number of cracks that appeared was determined using a microscope equipped with an image capture camera, using the Material Plus 4.2 software. The length of the cracks was measured at the jet exit area according to Figure 5.

The samples were cut from marble tiles with a thickness of 20 mm by complete cutting to a length of 100 mm and a width of 10 mm. At the exit of the jet, the cracks with a length greater than 0.1 mm were counted, passing the cut sample in front of the microscope. The methodology of experiment planning is based on statistical and graphical analysis.

The Taguchi method was used, by choosing the criterion to be optimized, reducing the number of cracks, then by establishing the factors influencing the process (Table 2), and their levels (the values they can take), after which the matrix of necessary experiments was chosen. Depending on this matrix, the experiments were performed, repeating each experiment 2 times, in order to reduce the random values. The Taguchi method is based on the analysis of the system’s response to factor variability, by introducing the signal/noise ratio (Y/N). The optimization of the system is done by choosing the configuration that has the maximum S/N value [37,38].

(6) $$\bar{y}=\frac{\sum_{i=1}^{n}y_{i}}{n}$$

where$\;\bar{y}$ is the arithmetic mean of the answers received, and *n* represents the number of tests performed with the same combination of parameters.

(7) $$s^{2}=\frac{\sum_{i=1}^{n}{\left(y_{i}-\bar{y}\right)}^{2}}{n-1}$$

where s^2^ is the dispersion of the system responses.

(8) $$\frac{S}{N}=-10log\left[s^{2}+{\bar{y}}^{2}\right]$$

In the graphical method used, were considered 3 fixed parameters, the other two taking the possible values in turn. In this way, the variability of two factors as well as the interaction between them influenced the responses of the system. This influence was shown graphically allowing conclusions to be drawn on the influence on the system.

## 4. Results and Discussion

The data obtained were summarized in Table 3, Table 4, Table 5, Table 6 and Table 7. Tests showing values of identical configurations were not repeated. This was done in order to maintain consistency in the interpretation of the results.

The diameter of the constant mixing tube (0.75 mm) was chosen in Table 3 and Table 4 because only the influence of pressure and stand-off distance, respectively abrasive granulation, was investigated. In Table 5, the research was directed at the influence of the pressure and diameter of the mixing tube. The pressure, because it has a great influence on the crack formation process, was chosen as the main factor, the influence of the other factors being researched together with the pressure change.

The graphs were drawn in the Minitab program, and show how the 4 parameters influence the appearance of cracks in the marble at hydro-abrasive jet cutting. Thus, it can be seen in all three graphs that with the increase of the pressure, from 180 MPa, to 250 MPa, the number of cracks doubles.

**Figure 6:**
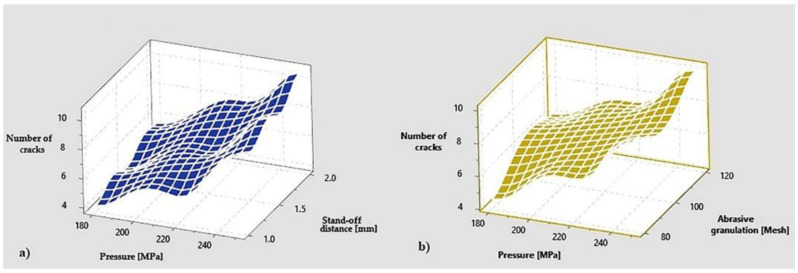
Dependence of number of cracks on: (**a**) pressure and stand-off distance; (**b**) pressure and abrasive granulation.

**Figure 7:**
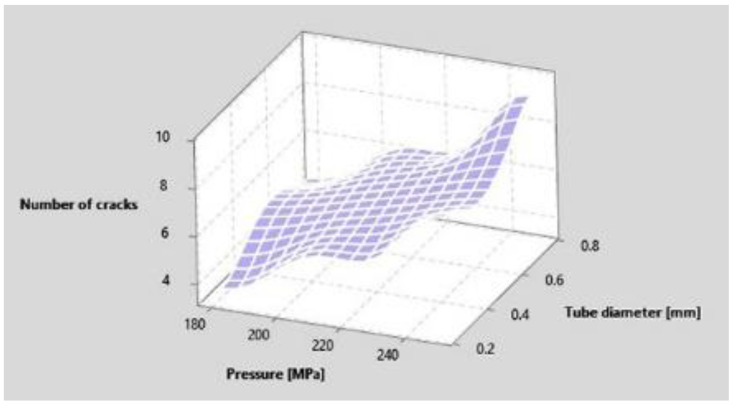
Dependence of number of cracks on pressure and tube diameter.

A smaller increase in the number of cracks is also registered for the other factors. Thus, the increase of the abrasive granulation, of the stand-off distance as well as the increase of the tube diameter lead to the increase of the number of cracks (Figure 6), with the mention that the least influence has the abrasive granulation and the biggest is the stand-off distance. Furthermore, increasing the diameter of the tube leads to an increase in the number cracks (Figure 7). The effect of the interaction between the other factors, being considered smaller, was no longer represented graphically. For Taguchi optimization, 4 factors with 2 levels were chosen using the values of the factors in Table 6, being made 4 samples with the same configuration of the factors. The registered values of the results are shown in Table 7.

**Figure 8:**
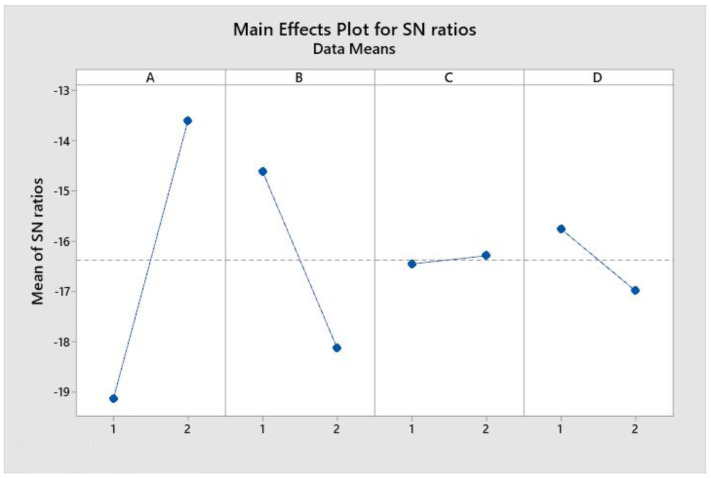
System optimization by configuring parameters, according to the Taguchi method.

In Minitab software were achieved the graphs representing the effects of each factor according to Figure 8.

As the Taguchi method involves maximizing the S/N ratio, the optimal configuration is A2, B1, C2, D1, (P = 180 MPa, d = 1 mm, G = 80 Mesh and D = 0.25 mm). It follows that optimizing the abrasive jet cutting of marble in order to reduce the number of cracks can be achieved not only by using low pressure but also by reducing stand-off distance, by working with a finer abrasive and by choosing a tube with a smaller diameter. Thus, working at low pressure P = 180 MPa, the cumulative influence of the other factors is 56% and at high pressure P = 250 MPa, the cumulative influence reaches 95%.

The repetition of the tests with common configurations was done to obtain the most accurate values, by calculating the arithmetic mean, and the number of experiments with different configurations was chosen according to the L8 experiment matrix corresponding to a number of 4 factors with 2 levels. Factorial statistical analysis can be extended to other parameters that influence the process.

## 5. Conclusions

Marble processing with the help of hydro-abrasive jet is a widespread process due to its great advantages. However, the results are not always as expected, especially due to the possibility of cracks. The cracking of the brittle materials and their jet cutting process was carefully studied and presented in the paper. From this study, it resulted that for the cracking of the marble in the abrasive jet cutting, so far, no concrete method has been applied to allow the choice of parameters in such a way as to optimize the process. Applying statistical analysis to the process, due to the variables involved, is the only way to optimize and has been applied in this research. Thus, the method of Taguchi fractional experiment plans was applied, allowing the choice of optimizable parameters. This paper provides users with a tool to optimize the results obtained by reducing rejections due to cracking.

It has been shown that:

- The increase of the pressure and of the stand-off distance, negatively influences the cracking of the marble;
- The diameter of the tube and the granulation of the abrasive have a smaller influence, which acts in the same way, by increasing them the possibility of cracking increases.

The Taguchi method allows the study of the influence of factors, taking into account the interaction between them. Applying the method can lead to finer workpieces, avoiding cracking due to thin walls. The method can be used to study the influence of other parameters such as the traverse speed, the angle of inclination of the hydro-abrasive jet, the gripping and immersion mode of the workpieces. In this way the optimization of the cutting with a hydro-abrasive jet of the marble is realized, reducing the negative effect of the variables due to the structure of the marble.

Thus, the relatively simple to apply Taguchi method leads to promising results in reducing the number of cracks when processing marble with a hydro-abrasive jet. Once the influence of a parameter is established, it can be modified accordingly. Of course, only the mentioned factors have been considered in this paper, but other factors may be involved in the processing. An example would be the cutting speed, the angle of attack of the surface or the use of another type of abrasive (diamond). Determining the influence of these factors can be made more difficult by users with more complex equipment—the use of a high-performance microscope to analyze experimental data. It is considered in future research and analysis of the influence of these parameters.

## Figures and Tables

**Table 1:** Inclusion analysis made with Material Plus software.

Inclusion-ASTM E112/E45-97				
Original image	Processed image 1		Processed image 2	
Inclusion types	Fine	Thick	Fine	Thick
Type A Analysis	0.00	0.00	0.00	0.00
Type B Analysis	0.00	1.00	1.00	0.00
Type C Analysis	0.50	2.00	0.50	2.00
Type D Analysis	1.50	6.50	1.50	4.50

**Table 2:** Parameters proposed for optimization.

Pressure P [MPa]	Stand-Off Distance d [mm]	Abrasive Granulation G [Mesh]	Tub Diam. D [mm]
180	1	80	0.25
220	1.5	120	0.75
250	2		

**Table 3:** Number of cracks depending on pressure and stand-off distance.

Pressure P [MPa]	Stand-Off Distance h [mm]	Abrasive Granulation G [Mesh]	Tub Diam. D [mm]	Number of Cracks		
				Test		
				1	2	$\bar{y}$
180	1	80	0.75	4	4	4
180	1.5	80	0.75	4	5	4.5
180	2	80	0.75	6	5	5.5
220	1	80	0.75	6	5	5.5
220	1.5	80	0.75	7	6	6.5
220	2	80	0.75	8	8	8
250	1	80	0.75	9	9	9
250	1.5	80	0.75	9	10	9.5
250	2	80	0.75	10	11	10.5

**Table 4:** Number of cracks depending on pressure and abrasive granulation.

Pressure P (Mpa)	Stand-Off Distance h (mm)	Abrasive Granulation G (Mesh)	Tub Diam. D (mm)	Number of Cracks		
				Test		
				1	2	$\bar{y}$
180	1.5	80	0.75	4	5	4.5
180	1.5	120	0.75	6	6	6
220	1.5	80	0.75	7	6	6.5
220	1,5	120	0.75	8	7	7.5
250	1.5	80	0.75	9	10	9.5
250	1.5	120	0.75	10	10	10

**Table 5:** Number of cracks depending on pressure and tube diameter.

Pressure P (Mpa)	Stand-Off Distance d (mm)	Abrasive Granulation G (Mesh)	Tub Diam. D (mm)	Number of Cracks		
				Test		
				1	2	$\bar{y}$
180	1.5	80	0.25	4	3	3.5
180	1.5	80	0.75	4	5	4.5
220	1.5	80	0.25	6	5	5.5
220	1.5	80	0.75	7	6	6.5
250	1.5	80	0.25	7	9	8
250	1.5	80	0.75	9	10	9.5

**Table 6:** Parameters proposed for Taguchi optimization.

Level	Pressure P [MPa]	Stand-Off Distance d [mm]	Abrasive Granulation G [Mesh]	Tub Diam. D [mm]
	A	B	C	D
1	250	1	120	0.25
2	180	2	80	0.75

**Table 7:** Taguchi experiment matrix.

	Pressure P [MPa]	Stand-Off Distance d [mm]	Abrasive Granulation G [Mesh]	Tub Diam. D [mm]	Number of Cracks						
					Test						
	A	B	C	D	1	2	3	4	$\bar{y}$	s	S/N
1	1	1	1	1	7	6	7	8	7	0.81650	−16.9461
2	1	1	2	2	8	7	9	9	8.25	0.95743	−18.3727
3	1	2	1	2	12	11	13	11	11.75	0.95743	−21.4223
4	1	2	2	1	10	11	10	8	9.75	1.25831	−19.8340
5	2	1	1	2	4	5	3	4	4	0.81650	−12.1748
6	2	1	2	1	3	3	4	4	3.50	0.57735	−10.9691
7	2	2	1	1	5	6	7	5	5.75	0.95743	−15.2827
8	2	2	2	2	6	7	5	7	6.25	0.95743	−15.9934

$\bar{y}$ is mean of results for same configuration, s is standard deviation and S/N is signal/noise ratio.

## Data Availability

The data presented in this study are available upon request from the corresponding author.

## References

[1] Štefek A., Raška J., Hlaváč L.M., Spadło S. (2021). Investigation of Significant Parameters during Abrasive Waterjet Turning. Materials.

[2] Spadło S., Bańkowski D., Młynarczyk P., Hlaváčová I.M. (2021). Influence of Local Temperature Changes on the Material Microstructure in Abrasive Water Jet Machining (AWJM). Materials.

[3] Karmiris-Obrata’nski P., Karkalos N.E., Kudelski R., Papazoglou E.L., Markopoulos A.P. (2021). On the Effect of Multiple Passes on Kerf Characteristics and Efficiency of Abrasive Waterjet Cutting. Metals.

[4] Melentiev R., Fang F. (2018). Recent advances and challenges of abrasive jet machining. CIRP J. Manuf. Sci. Technol..

[5] Arab P.B., Celestino T.B. (2020). A microscopic study on kerfs in rocks subjected to abrasive waterjet cutting. Wear.

[6] Torrubia P.L., Billingham J., Axinte D.A. (2016). Stochastic modelling of abrasive waterjet footprints using finite element analysis. Proc. R. Soc. A.

[7] Pawar P.J., Vidhate U.S., Khalkar M.Y. (2018). Improving the quality characteristics of abrasive water jet machining of marble material using multi-objective artificial bee colony algorithm. J. Comput. Sci. Eng..

[8] Rao R.V., Rai D.P., Balic J. (2017). A multi-objective algorithm for optimization of modern machining processes. Eng. App. Art. Int..

[9] Perec A. (2021). Multiple Response Optimization of Abrasive Water Jet Cutting Process using Response Surface Methodology (RSM). Proc. Comp. Sci..

[10] Natarajan Y., Kumar Murugesan P., Mohan M., Khan S.A.L.A. (2020). Abrasive Water Jet Machining process: A state of art of review. J. Manuf. Process..

[11] Liu X., Liang Z., Wen G., Yuan X. (2019). Waterjet machining and research developments: A review. Int. J. Adv. Man Technol..

[12] Zheng Y., He L. (2021). TBM tunneling in extremely hard and abrasive rocks: Problems, solutions and assisting methods. J. Cent. South Univ..

[13] Perec A. (2021). Research into the Disintegration of Abrasive Materials in the Abrasive Water Jet Machining Process. Materials.

[14] Wang F., Zhou D., Zhou X., Xiao N., Guo C. (2020). Rock Breaking Performance of TBM Disc Cutter Assisted by High-Pressure Water Jet. Appl. Sci..

[15] Hlaváč L.M., Annoni M.P.G., Hlaváčová I.M., Arleo F., Viganò F., Štefek A. (2021). Abrasive Waterjet (AWJ) Forces—Potential Indicators of Machining Quality. Materials.

[16] Cha Y., Oh T.-M., Hwang H.-J., Cho G.-C. (2021). Simple Approach for Evaluation of Abrasive Mixing Efficiency for Abrasive Waterjet Rock Cutting. Appl. Sci..

[17] Llanto J.M., Tolouei-Rad M., Vafadar A., Aamir M. (2021). Recent Progress Trend on Abrasive Waterjet Cutting of Metallic Materials: A Review. Appl. Sci..

[18] Liu S., Zhou F., Li H., Chen Y., Wang F., Guo C. (2020). Experimental Investigation of Hard Rock Breaking Using a Conical Pick Assisted by Abrasive Water Jet. Rock Mech. Rock Eng..

[19] Hlaváč L.M. (2021). Revised Model of Abrasive Water Jet Cutting for Industrial Use. Materials.

[20] Gupta V., Pandey P.M., Garg M.P., Khann R., Batra K.N. (2014). Minimization of kerf taper angle and kerf width using Taguchi’s method in abrasive water jet machining of marble. Proc. Mater. Sci..

[21] Joel C., Jeyapoovan T. (2021). Optimization of machinability parameters in abrasive water jet machining of AA7075 using Grey-Taguchi method. Mater. Today Proc..

[22] Perumal A., Kailasanathan C., Wilson V.H., Kumar T.S., Stalin B., Rajkumar P.R. (2021). Machinability of Titanium alloy 6242 by AWJM through Taguchi method. Mater. Today Proc..

[23] Balaji K., Siva Kumar M., Yuvaraj N. (2021). Multi objective taguchi–grey relational analysis and krill herd algorithm approaches to investigate the parametric optimization in abrasive water jet drilling of stainless steel. Appl. Soft. Comput..

[24] Barabas B., Deaconescu T. (2017). Researches regarding influence of traverse speed and stand-off distance to the roughness in AWJ process. Matec. Web Conf..

[25] Anstis G.R., Chantikul P., Lawn B.R., Marshall D.B. (1981). A Critical Evaluation of Indentation Techniques for Measuring Fracture Toughness I, Direct Crack Measurements. J. Am. Ceram. Society.

[26] Lawn B. (1993). Fracture of Brittle Solids.

[27] Le H., Wang H., Tang L., Ren X., Cai Y. (2021). Fracture of brittle solid material containing a single internal crack of different depths under three-point bending based on 3D-ILC. Eng. Fract. Mech..

[28] Conti P. Microstructures, textures and material properties of marble rocks; an introduction. Proceedings of the Geological Setting, Mineral Resources and Ancient Works of Samos and Adjacent Islands of the Aegean Sea.

[29] Nguyen T., Wang J. (2019). A review on the erosion mechanisms in abrasive waterjet micromachining of brittle materials. Int. J. Extrem. Manuf..

[30] Jafar R.H.M. (2013). Erosion and Roughness Modeling in Abrasive Jet Micro-Machining of Brittle Materials.

[31] Jafar R.H.M., Nouraei H., Emamifar M., Papini M., Spelt J.K. (2015). Erosion modeling in abrasive slurry jet micro-machining of brittle materials. J. Manuf. Process..

[32] Dun L., Thai N., Wang J., Huang C. (2020). Mechanisms of enhancing the machining performance in micro abrasive waterjet drilling of hard and brittle materials by vibration assistance. Int. J. Mach. Tools Manuf..

[33] Slikkerveer P.J., Bouten P.C.P., Veld F.H., Scholten H. (1998). Erosion and Damage by Sharp Particles. Wear.

[34] Zhang L., Ji R., Fu Y., Qi Y., Kong F., Li H., Tangwarodomnukun V. (2020). Investigation on particle motions and resultant impact erosion on quartz crystals by the micro-particle laden waterjet and airjet. Powder Technol..

[35] Qu H., Wu X., Liu Y., Feng Y., Tang S., Zhang S., Hu Y. (2020). Effect of shale mineralogy characteristics on the perforation performance and particle fragmentation of abrasive waterjet. Powder Technol..

[36] Hutchings I., Shipway P. (2017). Tribology, Friction and Wear of Engineering Materials.

[37] Taguchi G., Chowdhury S., Wu Y. (2004). Taguchi Quality Engineering Book.

[38] Deaconescu A., Deaconescu T. (2021). Response Surface Methods Used for Optimization of Abrasive Waterjet Machining of the Stainless Steel X2 CrNiMo 17-12-2. Materials.

